# DIGITAL COMMUNICATION AS COMPENSATION FOR INFREQUENT IN-PERSON CONTACT WITH GRANDCHILDREN DURING THE PANDEMIC

**DOI:** 10.1093/geroni/igad104.1446

**Published:** 2023-12-21

**Authors:** Xiaoyu Fu, Woosang Hwang, Merril Silverstein

**Affiliations:** Syracuse University, Syracuse, New York, United States; Texas Tech University, Lubbock, Texas, United States; Syracuse University, Syracuse, New York, United States

## Abstract

Maintaining interactive relationships with grandchildren represents an important and rewarding aspect of family life for grandparents. The goal of this study is to explore stylistic patterns of intergenerational contact with grandchildren including digital communication (texting, e-mail, video call, and social media interaction) and how they were associated with older grandparents’ psychological well-being during the COVID-19 pandemic. Using data from the 2021-22 survey of the Longitudinal Study of Generations (LSOG), the analysis applied latent class analysis to identify communication styles with grandchildren among 264 grandmothers and 174 grandfathers. We identified four communication styles for both grandmothers and grandfathers. Life satisfaction was relatively low and no different among grandparents with a detached style (little contact of any type) and those with a digitally connected style (little in-person contact but some digital contact). However, fully connected grandparents (digital contact and in-person contact) had significantly better life satisfaction than detached grandparents. Results suggest that digital communication with grandchildren did not compensate for the lack of face-to-face contact with grandchildren but complemented this more traditional form of contact. We conclude that digital contact with grandchildren provides added value above and beyond in-person contact for the psychological well-being of older grandparents.

